# Effects of Social Capital and Leisure Participation on Self-Rated Health of Urban Residents in Southwest China

**DOI:** 10.3389/fpubh.2021.763246

**Published:** 2021-11-01

**Authors:** XiaoYan Hu, MingWen Hu

**Affiliations:** ^1^School of Physical Education, North Sichuan Medical College, Nangchong, China; ^2^School of Physical Education, China West Normal University, Nangchong, China

**Keywords:** social capital, health promotion, self-rated health, leisure participation, exploratory analysis, leisure behavior

## Abstract

**Background:** Leisure provides opportunities for urban and rural residents to relax, recover their vitality, and improve their personal growth, development, and well-being. However, the impact of the leisure participation process, types, obstacles, participation motivation, and satisfaction on health is not very clear, especially the impact of leisure behavior on health, and is worthy of in-depth discussion.

**Objective:** The objective of this study was to explore the impact of social capital and leisure participation on the self-rated health of urban residents in China so as to provide an important reference for national health promotion activities.

**Methods:** the questionnaire on the relationship between social capital, leisure behavior, and self-rated health was compiled by ourselves. The residents participating in leisure and fitness in 25 residential fitness centers in Chengdu were investigated in the morning and evening, and the obtained data were processed by exploratory and confirmatory factor analysis.

**Results:** (1) Social capital had no direct influence on leisure hindrance; leisure motivation and leisure participation had no direct influence on self-rated health. (2) Leisure satisfaction has a direct positive impact on self-rated health, while leisure hindrance has a significant negative impact on self-rated health. (3) Social capital has a direct positive impact on leisure satisfaction, and social capital has a direct positive impact on self-rated health. Leisure satisfaction plays an intermediary role in the path of social capital affecting self-rated health, and the intermediary force exceeds the direct impact of social capital on self-rated health.

**Conclusion:** The effect of leisure satisfaction on self-rated health is higher than that of social capital, and it plays an intermediary role in the impact path of social capital on self-rated health. Therefore, how to make urban community residents with different backgrounds obtain leisure satisfaction through leisure activities is an important topic of national health promotion.

## Introduction

Contemporary social leisure activities have a strong impact on the life of urban residents. Leisure activities not only reflect the lives, politics, society, business, and religious systems of people but also provide people with relaxation, recovery of vitality, and improvement of personal growth, development, and happiness. Leisure is an important factor that determines the level of personal health. It can alleviate mental pressure, promote mental health, and reduce the occurrence of diseases. At the same time, it can also promote physical health, enhance physique, and improve resistance to diseases. Leisure also has the function of health investment, which can reduce personal medical expenses and reduce a medical burden. Human capital theory regards health of everyone as a kind of capital reserve, in which health capital is not only the most important human capital but also the main carrier of improving human capital. The accumulation of health capital can increase “healthy time” and improve labor productivity, and the accumulation of health capital is nothing more than positive leisure sports, diet and nutrition, and reverse medical care. Aminzadeh et al. (1) believe that leisure can not only improve personal happiness but also reduce life pressure and maintain physical and mental health. In exploring the context of a healthy society, Chemtob et al. (2) found that leisure participation can help people generate social support and personal self-determination to adjust to stress. Veenstra et al. (3) pointed out that proper participation in leisure activities can enhance cardiopulmonary fitness, exercise muscle strength, improve muscle tolerance, promote softness, strengthen the skeleton and control weight, etc.

In addition to leisure participation, social capital is also an important factor affecting individual health. Hall and Battaglio (4) believes that social capital is social connection and related norms and trust, which can promote coordination and cooperation of mutual interests. Social capital is an invisible resource. It helps individuals develop in work, family, community, and other fields, including education, employment, career promotion, happiness, health, and longevity. Many studies have shown that, with better social capital and better health, both individual family social capital and community social capital are important influencing factors on health, that is, individuals with good family interpersonal network and trust, reciprocal norms, and individuals with a high degree of the interpersonal network across the family will have better health (5–7). Some scholars also put forward different views. Aliev et al. (8) believe that women have a high rate of depression, which can be attributed in part to the reliance of women on trustworthy interpersonal relationships and the obligations brought about by such relationships. Therefore, women are less able to resist the loss of such support and will be affected when others in their own social network encounter adversity. Veenstra (9) also believes that social networks may become a channel for infectious diseases and unhealthy behaviors, worsen diseases, and thus have a negative impact on health. The research of Hyyppa and Maki (10) pointed out that the relationship between social capital and health is very weak, and even has a potential negative impact on health. It can be seen from the above that there is no consistent conclusion on whether social capital has a positive effect on health. Therefore, it is necessary to further explore the impact of social capital on health in this study.

The social relationship network of social capital is not formed naturally. It must be continuously produced and accumulated through some social activities, that is, through the participation of sports teams and artistic and cultural activities, the connection of members of different groups, and building social capital (4). Horolets et al. (11) pointed out that social capital is a by-product of social activities. The social capital generated by leisure activities promotes autonomy, trust, mutual assistance, and communication with others, and reduces mistrust and loneliness. Therefore, leisure activities can provide an important occasion for the composition of social capital. Kramer et al. (12) discussed the positioning of leisure in social capital and believed that leisure plays an important role in maintaining social capital in today's society. According to Lindstrom and Hanson (13), individuals participate in specific behaviors and seek specific relationships to obtain channels for resources (such as information, status, and wealth). Those resources are collectively shared by other social groups. Through this process of seeking and establishing relationships, individuals eventually become an integral part of a large social structure. The result is the growth of a common social capital structure, so social capital and leisure participation circulate and influence each other.

**Figure 1:**
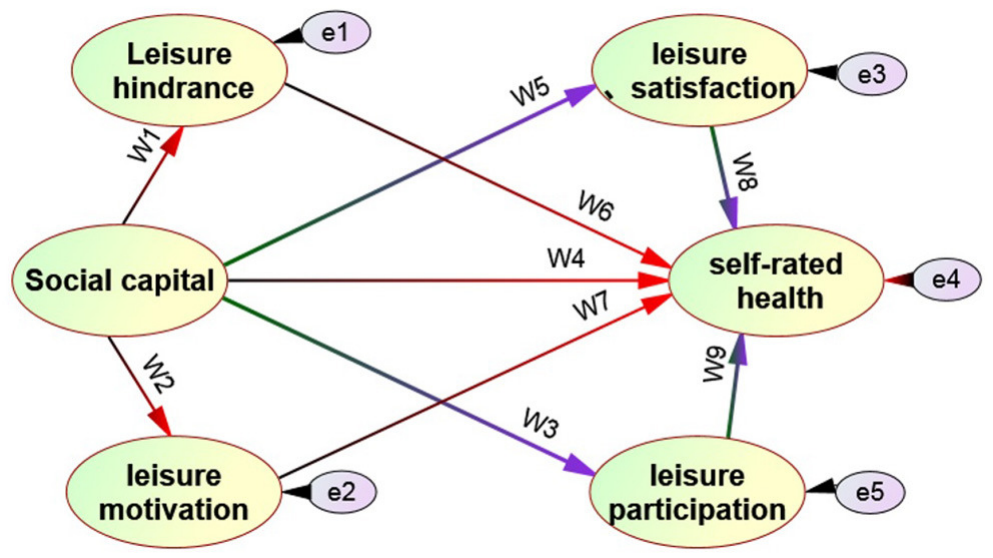
Hypothetical model of the relationship between social captial and leisure behavior on self-rated health.

Based on the literature of the above domestic and foreign scholars on leisure participation, social capital, and individual self-rated health, most studies take a single factor as the research variable, such as the relationship between leisure participation and health, the relationship between leisure types and health, the relationship between leisure sports obstacles and physical and mental health, the relationship between leisure motivation and health, the relationship between leisure satisfaction and health, etc (14–16). Few scholars have reported on the comprehensive exploration of social capital, leisure motivation, leisure obstacles, and health. Betkman et al. (17) proposed that leisure behavior may play a bridging role in the relationship between social capital and self-rated health, from the overall social structure factors to the causal process framework of personal physiological and mental health. This idea provides a verifiable theoretical model for this study on the relationship between social capital, leisure behavior, and health. Due to the lack of relevant research data, this study adopts the theoretical model of social network and health relationship structure, and uses empirical methods to explore the possible intermediary role of leisure behavior in the relationship between social capital and health from the perspective of sociology so as to explore the relationship model between social capital, leisure behavior, and self-rated health (see Figure 1 for the hypothetical model). Through the verification of the linear structure model, it is expected to have a further understanding of the antecedents and consequences variables affecting the leisure behavior of urban residents so as to provide an important reference for the national health promotion activities.

## Methods

### Respondents

In this study, 25 residential areas, such as bihualin, Hengye star garden, and Wuzhou garden in Chengdu, were officially tested as survey points. There are good fitness places in these areas. The researchers conducted a questionnaire survey on residents over the age of 25 who came to the community fitness venues for exercise at the two important time nodes of 6:30 a.m. and 7:00 p.m. Five investigators stayed in each residential area for 7 days. The whole research time began on March 6, 2021, and ended on May 15, 2021. A total of 2,843 valid sample data were obtained.

### Questionnaire Design

A structured questionnaire was designed, and was divided into five parts.

(1) Personal background information of subjects. It mainly includes gender, age, personal income, education level, and marital status.
(2) Social capital scale. It is mainly based on the scale (4, 18) prepared by Hall and Kaiser and modified according to the needs of this study, with a total of 30 items.
(3) Leisure motivation scale. It mainly refers to the leisure motivation scale developed by Roychowdhury (19), with a total of 32 items.
(4) Leisure participation scale. Mainly referring to the research of Brajsa-Zganec et al. (20) and YaoLin et al. (21), the leisure types are divided into 15 categories, namely outdoor activities, water activities, sports, music, dance, art, drama, craft, hobbies, intelligence, audiovisual, leisure, diet, folk activities, and social activities. Then, the factor analysis method is adopted for the 15 leisure types. Then, it is named according to the characteristics of the activity items included in the common factor as the measurement dimension of urban residential leisure participation.
(5)Leisure hindrance scale. It is mainly designed with reference to the dimension of leisure obstacles proposed by Raymore et al. (22) and Su Shiang et al. (23). The full scale has 21 items in total.
(6) Leisure satisfaction scale. It mainly refers to the leisure satisfaction scale developed by Huilin (24), which divides leisure satisfaction into six dimensions: psychological orientation, educational orientation, social orientation, relaxation orientation, biological orientation, and aesthetic orientation, with 24 items.
(7) Self-rated health scale. It mainly refers to Sokman L. physical and mental health scale (25) and SF-36 scale, with a total of 36 items. Likert scale five-point scoring scale was used for all six scales.

### Validity and Reliability of the Questionnaire

Before the formal survey, the test-retest reliability of the questionnaire shall be tested. Taking the three parks of Xinyizhou, Jinsha site, and Qinglong Lake in Chengdu as the survey points, and taking urban residents over the age of 25 as the objects, the citizens who came to the park to engage in leisure and fitness were investigated, and a total of 180 questionnaires were collected. The validity was tested by factor analysis (principal component analysis) and then rotated by the direct skew method. The factor load included in the factor item was not <0.4; The reliability test is based on Cronbach's α Coefficient. Amos measurement model structural validity and combination reliability were used as criteria.

Table 1 shows the following:

(1) Exploratory factor analysis showed that the Kaiser-Meyer-Olkin (KMO) values of six scales, including social capital, leisure motivation, leisure participation, leisure obstacles, leisure satisfaction, and self-rated health, reached a significant level, indicating that the six scales are suitable for factor analysis. According to the standard six scales with characteristic root >1, the number of common factors can be obtained as 4, 4, 3, 3, 5, and 3, respectively; according to the standard, the factor load is not <0.4, and the common factor characteristics corresponding to the six scales are as follows:
  (a) The four common factors of social capital scale were named social network, social norms, social participation, and social trust. The corresponding explanatory variation was 38.25, 12.14, 9.24, and 8.11%, respectively, and the cumulative explanatory variation was 67.74%. The number of items included in the four factors is 5, 6, 6, and 6, respectively (23 questions in total, 7 deleted).
  (b) The four common factors of the leisure motivation scale were named competency proficiency, intelligence, stimulus avoidance, and sociality, respectively. The explanatory variation was 30.21, 20.69, 14.36, and 10.17%, respectively, and the cumulative explanatory variation was 79.43%; the number of items included in the four common factors is 6, 5, 5, and 5, respectively (21 questions in total, 11 deleted).
  (c) The three common factors of leisure participation scale were named outdoor sports type, artistic activity type, and daily leisure type, respectively. The explanatory variation was 30.56, 21.78, and 15.21%, respectively, and the cumulative explanatory variation was 67.75%; the number of items included in the three common factors is 3, 6, and 6, respectively (15 questions in total, all reserved).
  (d) The three common factors of the leisure hindrance scale were named interpersonal hindrance, structural hindrance, and personal internal hindrance, respectively. The explanatory variation was 34.55, 18.21, and 10.44%, and the cumulative explanatory variation was 63.20%; the number of items included in the three common factors is 6, 6, and 5, respectively (17 questions in total, 4 deleted).
  (e) The five common factors of the leisure satisfaction scale were named education orientation, aesthetic orientation, physical orientation, psychological orientation, and relaxation orientation, respectively. The explanatory variation was 35.36, 24.17, 13.65, 8.24, and 10.44%, and the cumulative explanatory variation was 87.42%; the number of items included in the five common factors is 5, 4, 5, 4, and 4, respectively (22 questions in total, 2 deleted).
  (f) Self-rated health scale. Three common factors can be extracted and named social health, mental health, and physical health, respectively. The explanatory variation is 38.27, 21.55, and 17.14%, respectively, and the cumulative explanatory variation is 76.96%; the number of items included in the three common factors is 9, 10, and 8, respectively (27 questions in total, 8 deleted).
(2) In the six scales measurement models of social capital, leisure motivation, leisure participation, leisure hindrance, leisure satisfaction, and self-rated health, the common factor combination reliability of each scale is more than 0.80 (higher than the standard of 0.60) and Cronbach α coefficients of all are above 0.75 (higher than the standard of 0.60); the test results of six measurement models show that, among the overall evaluation indexes, NRFI are 0.96, 0.91, 0.94, 0.90, 0.94, and 0.95, respectively (standard nrfi ≥0.90); CFI was 0.91, 0.93, 0.91, 0.93, 0.91, and 0.94, respectively (standard CFI ≥ 0.90); RMSEA is 0.05, 0.02, 0.06, 0.07, 0.03, and 0.06, respectively (standard RMSEA ≤ 0.08); SRMR is 0.04, 0.02, 0.01, 0.03, 0.04, and 0, respectively (standard SRMR ≤ 0.05). These indicators fully show that the six scales have good reliability and validity.

**Table 1:** A statistical table of validity reliability (quality) test of measurement scale.

	**Dimension name**	**Kmo and** **Bartlett test**	**Number** **of item**	**Explained** **variation %**	**Progressive interpretation** **variance %**	**Composite reliability** **CR**	**Cronbach** **α coefficient**
Social capital scale	Social network	KMO = 0.87;*P* < 0.05	5	38.25	38.25	0.84	0.85
	Social norms		6	12.14	50.39	0.87	0.81
	social participation		6	9.24	59.63	0.85	0.80
	Social trust		6	8.11	67.74	0.83	0.86
Measurement model verification results: NRFI = 0.96, CFI = 0.91, RMSEA = 0.05, SRMR = 0.04							
Leisure motivation scale	Competence proficiency	KMO = 0.84;*P* < 0.05	6	30.21	30.21	0.81	0.79
	Intelligence		5	20.69	54.90	0.86	0.88
	Stimulus avoidance		5	14.36	69.26	0.85	0.80
	Sociality		5	10.17	79.43	0.83	0.79
Measurement model verification results: NRFI = 0.91, CFI = 0.93, RMSEA = 0.02, SRMR = 0.02							
		*P* = 0.000					
Leisure participation scale	Outdoor sports style	KMO = 0.83;*P* < 0.05	3	30.56	30.56	0.83	0.80
	Artistic activity pattern		6	21.78	52.34	0.84	0.81
	Daily leisure style		6	15.21	67.75	0.87	0.84
Measurement model verification results: NRFI = 0.94, CFI = 0.91, RMSEA = 0.06, SRMR = 0.01							
Leisure hindrance scale	Interpersonal hinderance	KMO = 0.86;*P* < 0.05	6	34.55	34.55	0.82	0.90
	Structural hinderance		6	18.21	52.76	0.85	0.87
	Internal hinderance		5	10.44	63.20	0.88	0.91
Measurement model verification results: NRFI = 0.90, CFI = 0.93, RMSEA = 0.07, SRMR = 0.03							
Leisure satisfaction scale	Educational satisfaction	KMO = 0.91;*P* < 0.05	5	35.36	35.36	0.89	0.89
	Aesthetic satisfaction		4	24.17	59.53	0.87	0.83
	Physiological satisfaction		5	13.15	73.03	0.80	0.85
	Psychological satisfaction		4	8.24	81.27	0.84	0.82
	Relaxation satisfaction		4	6.15	87.42	0.88	0.90
Measurement model verification results: NRFI = 0.94, CFI = 0.91, RMSEA = 0.03, SRMR = 0.04							
Self rated health scale	Social health	KMO = 0.84;*P* < 0.05	9	38.27	38.27	0.89	0.85
	mental health		10	21.55	59.82	0.87	0.91
	Physical health		8	17.14	76.96	0.90	0.83
Measurement model verification results: NRFI = 0.95, CFI = 0.94, RMSEA = 0.06, SRMR = 0.00							

### Mathematical Statistics

Two main statistical analysis software packages, such as SPSS17.0 and almost 21.0, were used; descriptive analysis, correlation analysis, regression analysis, factor analysis, and structural equation model were used to statistically process the data. The significance level of all indicators is set as α = 0.05.

## Results

### Demographic Characteristics of Sports Participation of Middle-Aged Urban Residents in Chengdu

Table 2 shows the following:

(1) There were significant gender differences in sports participation behavior (x^2^ = 61.38, *P* = 0.000), which showed that the proportion of female regular athletes was significantly higher than that of male; (2) There was an obvious age difference in sports participation behavior (x^2^ = 141.21, *P* = 0.000), which showed that those older than 45 had a higher proportion of regular sports participation; (3) There was a significant educational difference in sports participation behavior (x^2^ = 411.31, *P* = 0.000), which showed that the proportion of regular sports participation was higher among highly educated people; (4) Exercise participation behavior was affected by work type (x^2^ = 120.00, *P* = 0.000), which showed that sitting workers had a higher proportion of regular exercise; (5) Sports participation behavior was affected by occupational types (x^2^ = 47.14, *P* = 0.000), which shows that the proportion of regular sports of cadres (including teachers) in organs and institutions was higher;(6) Sports participation behavior was not affected by family status (x^2^ = 1.38, *P* = 0.710 > 0.05).

**Table 2:** Analysis of social and demographic data of regular and irregular athletes (overall effective *n* = 2,843).

		**Irregular exerciser (%)** **(***N*** = 1,834)**	**Regular exerciser (%)** **(***N*** = 1,009)**	**X^2^**	***P***
Gender	Male	1025 (55.9)	409 (40.5)	61.38	0.000
	Female	809 (44.1)	600 (59.5)		
Age	≤ 40 years old	363 (19.8)	322 (31.9)	141.21	0.000
	41–45 years old	858 (46.8)	247 (24.5)		
	>45years old	613 (33.4)	440 (43.6)		
Cultural level	Primary school and below	326 (17.8)	122 (12.1)	411.31	0.000
	Middle school	943 (51.4)	206 (20.4)		
	College and undergraduate	500 (27.3)	506 (50.1)		
	Master degree or above	65 (3.5)	175 (17.3)		
Type of work	Sitting type	464 (25.3)	445 (44.1)	124.00	0.000
	Sitting or standing type	609 (33.2)	314 (31.1)		
	Goods to be moved	761 (41.5)	250 (24.8)		
Occupation type	Occupation 1	903 (49.2)	601 (59.6)	47.14	0.000
	Occupation 2	612 (33.4)	248 (24.6)		
	Occupation 3	154 (8.4)	109 (10.8)		
	Occupation 4	165 (9.0)	51 (5.1)		
Family status	Two-parent family	1,355 (73.9)	739 (73.2)	1.38	0.710
	Single-parent family	479 (26.1)	270 (26.8)		

*Occupation 1: Cadres of government organs and institutions (including teachers); Occupation 2: ordinary workers and service practitioners; Occupation 3: technicians and managers of private or national enterprises; 4: Other occupations (migrant workers and unemployed)*.

### Initial Model Validation of the Impact of Social Capital and Leisure Behavior on Self-Rated Health

This part takes social capital as the self-variable, leisure behavior (including leisure motivation, leisure participation, leisure hindrance, and leisure satisfaction) as the intermediary variable, and self-rated health of residents as the dependent variable to explore whether social capital will affect health through the intermediary effect of leisure behavior.

Table 3 shows the following:

(1) From the quality of the model structure. The three indicators of single variable reliability, potential variable combination reliability, and potential variable average variation extraction showed that the single variable reliability was more than 0.55 (exceeding the standard of 0.5), the potential variable combination reliability was more than 0.70 (exceeding the standard of 0.6), and the potential variable average variation extraction was more than 0.75 (exceeding the standard of 0.5). Therefore, this research model has quite ideal internal quality.
(2) In terms of absolute adaptation index, X^2^ = 587.14, DF = 374, X^2^/DF = 1.57 < 3, which meets the standard. In addition, both RMSEA = 0.021 (<0.1 standard) and RMR = 0.035 (<0.1 standard) meet the standard, which means that the overall model fits better. In addition, GFI = 0.920 (>0.9 standard) and AGFI = 0.915 (>0.9 standard) meet the standard.
(3) In terms of the value-added adaptation index, the closer the value is to 1, the better the adaptation degree is. Generally speaking, >0.9 means that the adaptation degree is excellent. In this study, the four value-added indexes of NFI, nnfi, IFI, and RFI are 0.924, 0.915, 0.933, and 0.942, respectively, which are greater than the standard of 0.9, indicating excellent adaptability.
(4) In terms of parsimony adaptation, this study takes CN, PNFI, PGFI, and AIC as indicators, and the obtained CN = 258.63, up to 200, indicating that the model can properly reflect the sample data; Pnfi = 0.726 and pgfi = 0.668 are >0.5, which means that the model is a simplified model. In addition, AIC = 815.14, which is significantly less than the AIC value of independent mode, meets the standard that the AIC of the theoretical model must be less than the AIC value of independent mode. In conclusion, the model obtained in this study has an ideal overall fit with the observed data, and the measurement model in this study is a simplified model.

**Table 3:** Parameter estimation and internal quality inspection statistics of the structural model validation analysis.

**Overall model quality index**	**social capital**				**Leisure motivation**				**Leisure hindrance**		
	**X_1_**	**X_2_**	**X_3_**	**X_4_**	**M_1_**	**M_2_**	**M_3_**	**M_4_**	**L_1_**	**L_2_**	**L_3_**
Single variable reliability	0.71	0.66	0.59	0.71	0.68	0.73	0.60	0.74	0.59	0.69	0.66
composite reliability	0.75	0.78	0.70								
Average variance extraction	0.80	0.81	0.79								
	**Leisure satisfaction**				**Leisure participation**				**Self rated health**		
	**G_1_**	**G_2_**	**G_3_**	**G_4_**	**G_5_**	**K_1_**	**K_2_**	**K_3_**	**Y_1_**	**Y_2_**	**Y_3_**
Single variable reliability	0.73	0.64	0.61	0.72	0.68	0.75	0.70	0.61	0.68	0.61	0.58
composite reliability	0.81	0.74	0.73								
Average variance extraction	0.82	0.87	0.77								

*Chi square value X^2^ = 587.14, df = 374, X^2^/df = 1.57, P = 0.19; GFI = 0.920 (≥0.9), AGFI = 0.915 (≥0.9), RMSEA = 0.021 (<0.1), RMR = 0.035 (<0.1), NFI = 0.924 (≥0.9), CFI = 0.974 (≥0.9), RFI = 0.942 (≥0.9), IFI = 0.933 (≥0.9), NNFI = 0.915 (≥0.9), RFI = 0.951 (≥0.9); CN = 258.63, PNFI = 0.726 (≥0.5); PGFI = 0.668 (≥0.5), AIC = 815.14*.

*X_1_, X_2_, X_3_, and X_4_ represent social networks, social participation, social norms, and social trust; M_1_, M_2_, M_3_, and M_4_ represent stimulus avoidance, competence proficiency, sociality, and intelligence, respectively; L_1_, L_2_, and L_3_ represent interpersonal hindrance, structural hindrance, and internal hindrance, respectively; G_1_, G_2_, G_3_, G_4_, and G5, respectively, represent education satisfaction, aesthetic satisfaction, physiological satisfaction, psychological satisfaction, and relaxation satisfaction; K_1_, K_2_, and K_3_ represent outdoor sports type, artistic sports type, and daily sports type, respectively; Y_1_, Y_2_, and Y_3_ represent social health, mental health, and physical health, respectively*.

### Initial Structural Model Suitability Analysis

**Figure 2:**
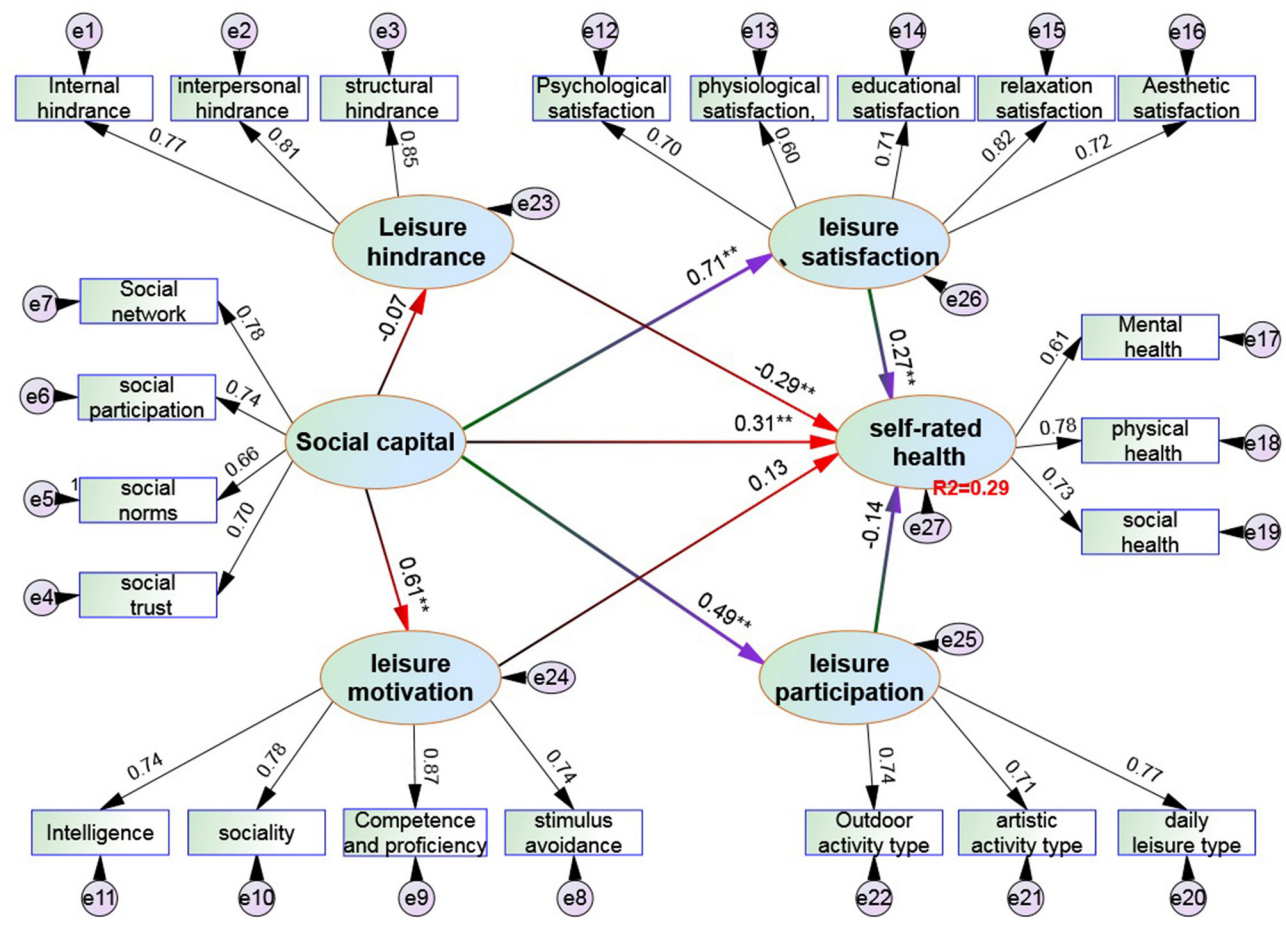
Structural equation model verification of the impact of social capital and leisure behavior on self-rated health. ^“*, **^, and ^***”^ represent the significant levels of 0.05, 0.01, and 0.001, respectively.

Figure 2 and Table 4 show the following:

(1) In the factor load among the potential variables of the measurement item, the social capital dimension (X_1_-X_4_) is λ; values were 0.78, 0.74, 0.66, and 0.70 (all higher than the standard value of 0.5); leisure hindrance dimension (L_1_-L_3_) λ values were 0.77, 0.81, and 0.75 (all higher than the standard value of 0.5); leisure motivation dimension (M_1_-M_4_) λ values were 0.74, 0.87, 0.78, and 0.74 (all higher than the standard value of 0.5); leisure satisfaction dimension (G_1_-G_5_) λ values were 0.71, 0.72, 0.60, 0.71, and 0.82 (all higher than the standard value of 0.5); leisure participation dimension (K_1_-K_3_) λ values were 0.74, 0.71, and 0.77 (all higher than the standard value of 0.5); of self-rated health dimension (Y_1_-Y_3_) λ values were 0.73, 0.78, and 0.61 (all higher than the standard value of 0.5). These results show that the hypothetical model meets the basic adaptation test.
(2) This study verified nine relationships. From the perspective of social capital → leisure hindrance, the path coefficient is −0.07, *p* >0.05, that is, social capital has no influence on leisure hindrance; from the impact of social capital → leisure motivation, the path coefficient is 0.61^**^, reaching a significant level of 0.01, indicating that social capital has a positive impact on leisure motivation; in terms of the impact of social capital → leisure satisfaction, the path coefficient is 0.71^**^, reaching a significant level of 0.01, indicating the significant positive impact of social capital on leisure satisfaction; from the impact of leisure satisfaction *n* → self-rated health, the path coefficient is 0.27^*^, reaching a significant level of 0.05, indicating that leisure satisfaction has a significant positive impact on self-rated health; from the impact of social capital → self-rated health, the path coefficient is 0.31^**^, *P* = 0.00 <0.05, indicating that social capital has a significant positive impact on self-rated health; from the impact of social capital → leisure participation, the path coefficient is 0.49^**^, reaching a significant level of 0.01, indicating that social capital has a significant positive impact on leisure participation; from the impact of leisure hindrance → self-rated health, the path coefficient is −0.29^**^, reaching a significant level of 0.01, indicating that leisure hindrance has a significant negative impact on self-rated health; from the impact of leisure motivation → self-rated health and leisure participation → self-rated health, the path coefficients are 0.13, −0.14, and the corresponding *P* is >0.05. Therefore, leisure motivation and leisure participation have no impact on self-rated health.

**Table 4:** Statistics of structural fitness test of the overall sample model.

**Causal relationship**	**Path** **coefficient β**	**Test and significance level**	**causal relationship**	**Path coefficient β**	**Test and significance level**
Social capital → Leisure hindrance	−0.07	*P* = >0.05; Unsupported	social capital → Leisure participation	0.49^**^	*P* = <0.01; Support
Social capital → Leisure motivation	0.71^**^	*P* = <0.01; Support	Leisure hindrance → Self rated health	−0.37^**^	*P* = <0.01; Support
Social capital → Leisure satisfaction	0.71^**^	*P* = <0.01; Support	Leisure motivation → Self rated health	0.13	*P* = >0.05; Unsupported
Social capital → Self rated health	0.27^**^	*P* = <0.05; Support	Leisure participation → Self rated health	−0.14	*P* = >0.05; Unsupported
			Leisure satisfaction → Self rated health	0.27^*^	*P* = <0.05; Support

^“*”, “**”, “***”^ represent the significant levels of 0.05, 0.01, and 0.001, respectively.

### Analysis of the Final Model of Social Capital and Leisure Behavior on Self-Rated Health

Based on the above structural model of social capital and leisure behavior (including motivation, hindrance, participation, and satisfaction) affecting self-rated health, the regression coefficients of social capital on leisure hindrance, leisure motivation on self-rated health, and leisure participation on self-rated health have not reached a significant level. Therefore, only the leisure motivation, leisure participation, and the leisure hindrance variables were deleted to ensure leisure satisfaction, and the fitness test was carried out again.

**Figure 3:**
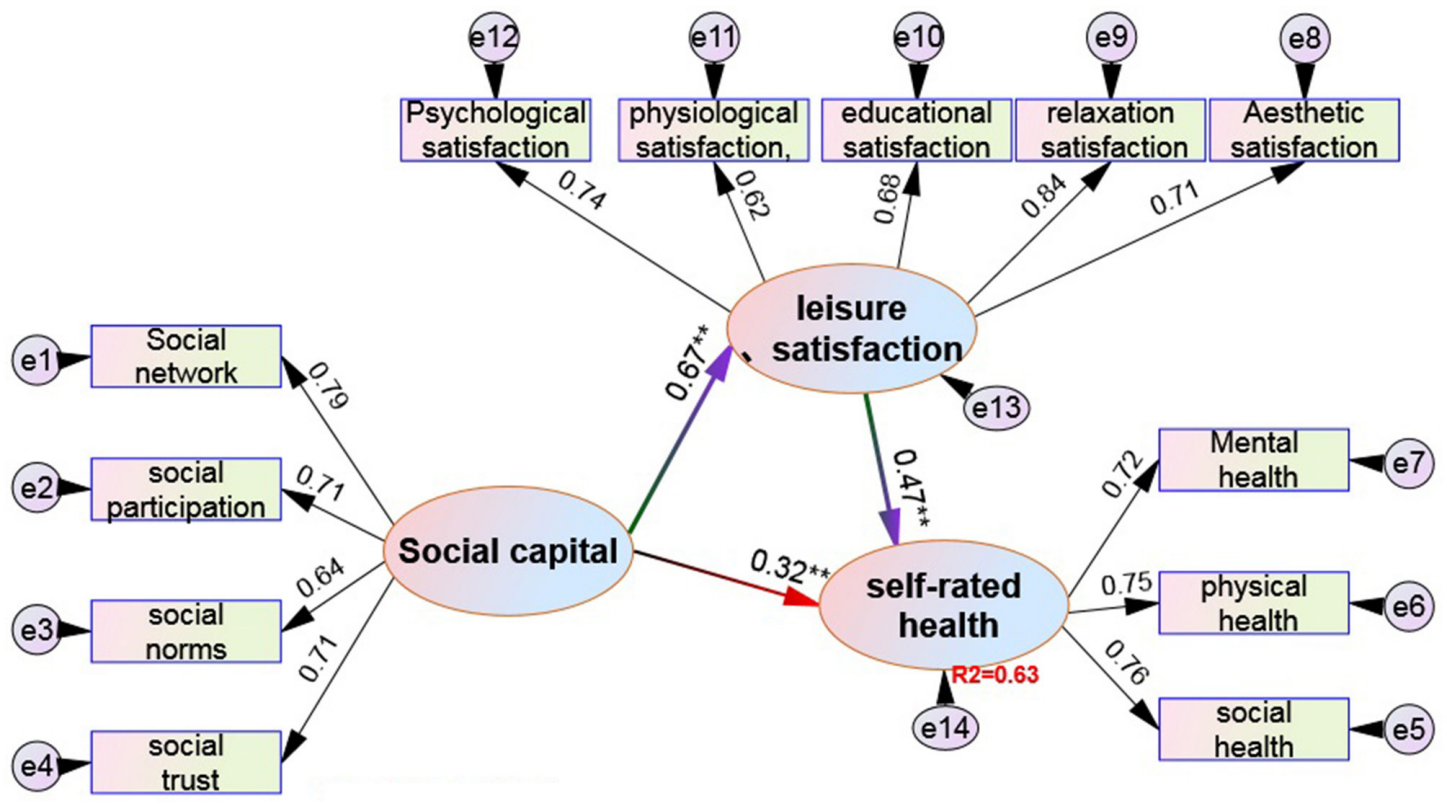
Validation of the relationship model between social capital, leisure satisfaction, and self-rated health. ^“*, **^, and ^***”^ represent the significant levels of 0.05, 0.01, and 0.001, respectively.

Table 5 and Figure 3 show the following:

(1) In terms of model structure quality, the reliability of a single variable is more than 0.65 (exceeding the standard of 0.5), the reliability of the combination of potential variables is more than 0.74 (exceeding the standard of 0.6), and the average variation extraction amount of potential variables is more than 0.82 (exceeding the standard of 0.5). Therefore, the model structure in Figure 3 has quite ideal internal quality. (2) In terms of absolute adaptation index, X^2^ = 85.58, DF = 68, X^2^/DF = 1.26 < 3, in line with the standard. In addition, RMSEA = 0.019 (less than the 0.1 standard) meets the standard, which means that the overall model fits better. In addition, GFI = 0.942 (>0.9 standard) and AGFI = 0.933 (greater than the 0.9 standard) meet the standard. (3) In terms of value-added adaptation indicators, the four value-added indexes of NFI, NNFI, IFI, and RFI are 0.930, 0.941, 0.928, and 0.935, respectively, which are all >0.9 standard, indicating excellent adaptability. At last, from the perspective of structural adaptation in the model: 1) social capital dimension (X_1_-X_4_) λ values were 0.79, 0.71, 0.64, and 0.71 (all higher than the standard value of 0.5); Leisure satisfaction dimension (G_1_-G_5_) λ values were 0.68, 0.71, 0.74, 0.62, and 0.84 (all higher than the standard value of 0.5); of self-rated health dimension (Y_1_-Y_3_) λ values were 0.76, 0.75, and 0.72 (all higher than the standard value of 0.5). These results show that the hypothetical model meets the basic adaptation test.

**Table 5:** Parameter estimation and internal quality inspection statistics of the structural model validation analysis.

**Overall model quality index**	**Social capital**				**Leisure satisfaction**					**Self rated health**		
	**X_1_**	**X_2_**	**X_3_**	**X_4_**	**G_1_**	**G_2_**	**G_3_**	**G_4_**	**G_5_**	**Y_2_**	**Y_2_**	**Y_3_**
Single variable reliability	0.75	0.71	0.68	0.73	0.70	0.74	0.65	0.73	0.66	0.74	0.70	0.73
Composite reliability	0.78				0.81				0.74			
Average variance extraction	0.82				0.83				0.84			
Social capital → leisure satisfaction, Standardized path coefficient β = 0.67^**^, supporting the hypothesis; Social capital → Self rated health, Standardized path coefficient β = 0.32^**^, supporting the hypothesis; Leisure satisfaction → self-rated health, Standardized path coefficient β = 0.47^**^, supporting the hypothesis.												

*Chi square value X^2^ = 85.58, df = 68, X^2^/df = 1.26, P = 0.27; GFI = 0.942 (≥0.9), AGFI = 0.933 (≥0.9), RMSEA = 0.019 (<0.1), NFI = 0.924(≥0.9), CFI = 0.974 (≥0.9), RFI = 0.942 (≥0.9), IFI = 0.928 (≥0.9), NNFI = 0.941 (≥0.9), RFI = 0.935 (≥0.9)*.

^“*”, “**”, “***”^ represent the significant levels of 0.05, 0.01, and 0.001, respectively.

The mediating role of leisure satisfaction is explored and calculated according to the bootstrap method. In this study, non-parametric percentile bootstrap was used to test the significance of mediating effect. The original data were sampled 1,000 times, and the 95% CI was estimated. First, it is judged that the indirect effect does not contain 0 within the 95% CI and reaches a significant level, indicating that there is an intermediary effect. At this time, if the direct effect contains 0 within the 95% CI, it means that the direct effect is not significant and is a complete intermediary effect; if the indirect effect and the direct effect do not include 0 in the 95% CI, both reach a significant level, and the total effect does not include 0 in the 95% CI, reaching a significant level; it is a partial intermediary effect.

It is directly derived from the structural equation model that the indirect influence of social capital on self-rated health is equal to 0.31^**^ (0.67 × 0.47), reaching a significant level, with a CI of 0.195–0.574, which, obviously, does not contain zero, while the direct influence of social capital on self-rated health = 0.32^**^, which is also very significant, with a CI of 0.177–0.563, which, obviously, does not contain zero, and the total effect = 0.63^**^, with a CI of 0.356–0.903, which also does not contain zero. Therefore, it can be judged that leisure satisfaction plays a partial intermediary role between social capital and self-rated health. From the overall influence of social capital and leisure satisfaction on self-rated health, the direct influence of leisure satisfaction on self-rated health (22% = 0.47 × 0.47) is significantly higher than the direct influence of social capital (10% = 0.32 × 0.32), plus the intermediary influence of leisure satisfaction (31%). Therefore, the total influence of social capital and leisure satisfaction on self-rated health is 63%.

## Discussion

The relationship between social capital and health can be traced back to the late Nineteenth century. Gold et al. (26) found that social trust and group participation are negatively correlated with mortality. Income inequality leads to low investment in social capital, which further has a negative impact on health; Sundquist et al. (27) found that personal factors, such as low income, low education, and smoking, are highly related to self-rated health, and low social capital is an important factor causing poor self-rated health; Boen et al. (28) pointed out that the mortality of people with strong social networks is only one-half to one-third of that of people with weak social networks, and personal social capital is related to self-worth, self-efficacy, and the ability to master life; Firouzbakht et al. (29) found that social capital has a positive influence. Both human capital and social capital are factors explaining health variation. Among them, social capital plays a higher role in improving physical and mental health than human capital. Although most studies reveal the positive effect of social capital on health, the research results of some scholars do not support this view. Miyamoto et al. (30) pointed out that social capital variables, such as trust, community participation, and citizen participation, have no significant impact on personal health. In short, in the past decade, the impact of social capital on self-rated health of residents has been discussed by many scholars. At the same time, the positive effect of social capital on health has also been confirmed by most studies. The results of this study show that social capital has a significant positive impact on self-rated health (with an explanatory variation of 10%), which is similar to many previous research results. However, from social capital to overall environmental factors, and from social capital to personal health, the causal link and the intermediate mechanism are quite complex, and there is a lack of convincing research results so far.

From the perspective of participation of social capital in leisure activities, Kim et al. (31) found that social capital and socioeconomic status can be used as the main predictors of participating in physical leisure activities. From the perspective of social capital, the difference in socioeconomic status of physical leisure activities is caused by the difference of social capital, that is, social capital is the intermediary factor of socioeconomic status and physical leisure activities. Loch et al. (32) found that social capital is an important factor affecting participation in leisure activities. Good family interaction and high-quality interpersonal network with friends and colleagues contribute to the occurrence of leisure activities. Zhang et al. (33) used tracking research to find that individuals who participated in more associations engaged in leisure activities, such as pure leisure, family planning activities, and voluntary work in a wider range and higher frequency. At the same time, the closer the social distance with your best friend, the easier it is to participate in leisure activities. The farther the social distance with your best friend, the less leisure activities. This study found that social capital has a significant positive impact on leisure activity participation (the explanatory variation is 24%), which supports the previous research views of scholars. This study decomposes leisure behavior into four aspects: leisure motivation, leisure hindrance, leisure participation, and leisure satisfaction. However, it is found that social capital has no direct impact on leisure hindrance. This finding is the same as the study of Pablo (34), that is, social capital has no significant impact on leisure hindrance. This study found that the path standardization coefficient of the impact of social capital on leisure satisfaction is large, reaching a very significant level, which means that social capital has a direct impact on leisure satisfaction. This result is the same as that of Briki (35), that is, social capital also has a direct impact on leisure satisfaction.

Previous scholars lack systematic and integrated research on the impact of sports and leisure on health. Lifshitz-Vahavt et al. (36) decomposed leisure benefits into physiological, economic, social, and psychological benefits. The results showed that physical leisure activities had a direct positive impact on physical health, such as reducing mortality, obesity, cardiovascular disease, etc. Rubio et al. (37) pointed to a survey that participation in leisure activities is negatively correlated with the anxiety and depression of unemployed and low-income women. The author believes that participation in leisure activities is conducive to maintaining mental health for women in disadvantageous living conditions. According to the survey of Shandra (38), life stress is positively correlated with the symptoms of mental and physiological diseases and negatively correlated with cognitive health, and points out that leisure partnership can alleviate the impact of life stress on the symptoms of mental diseases. This study found that leisure participation had no significant effect on self-rated health of residents, which was inconsistent with the previous research results, and the underlying reasons need to be further discussed in the follow-up.

By removing leisure motivation, leisure obstacles, and leisure participation from the original model, the modified model of social capital, leisure satisfaction, and self-rated health is obtained. The results show that the path coefficient of leisure satisfaction on self-rated health is 0.47^**^, reaching a significant level, indicating that leisure satisfaction has a direct impact on self-rated health. At the same time, the path coefficient of the influence of social capital on self-rated health is 0.32^**^, which also reaches a significant level. However, after considering the mediating role of leisure satisfaction, the influence of social capital and leisure satisfaction on self-rated health is up to 63%. This result shows that leisure satisfaction plays a very strong mediating role in the impact of social capital on self-rated health, which supports the research conclusions of Betkman and other scholars (17, 39), that is, the process model of social structural factors → social network → psychological social mechanism → healthy behavior approach → self-rated health. The overall social structural factors will limit or shape the structure and characteristics of the social network, and social network provides an opportunity to establish an individual psychological social mechanism, which affects self-rated health of people through some healthy behaviors. This study decomposes leisure behavior into four potential variables, hoping to fully and deeply reveal the relevant mechanism of leisure behavior on health promotion, but the study only finds that leisure satisfaction plays an intermediary role between social capital and self-rated health, which shows that leisure satisfaction is an individual through motivation, demand, and evaluation, and can expect leisure quality and bring individual demand satisfaction or overall evaluation satisfaction due to motivation, which may have included the evaluation of previous leisure motivation, leisure participation, and leisure obstacles, so it has a significant impact effect. Therefore, on the whole, in the influence model of social capital and leisure behavior on self-rated health, although leisure behavior only has a significant effect on leisure satisfaction, it highlights that leisure satisfaction is a key factor in the influence of social capital on self-rated health.

## Conclusion

(1) Among the four factors, leisure behavior, leisure motivation, and leisure participation have no direct impact on self-rated health; leisure satisfaction has a significant direct positive impact on self-rated health, while leisure hindrance has a significant direct negative impact on self-rated health. Social capital has no direct impact on leisure barriers but has a significant direct impact on leisure satisfaction, leisure motivation, and leisure participation.
(2) The direct effect of social capital on leisure satisfaction is significantly higher than that of social capital on self-rated health. Leisure satisfaction is established as an intermediary factor between social capital and self-rated health, and the intermediary force greatly exceeds the direct impact of social capital and leisure satisfaction on self-rated health.

## Suggestions

(1) The results show that social capital can positively affect health through the intermediary effect of leisure satisfaction, and it is found that the effect of leisure satisfaction on health is significantly higher than that of social capital. The content of leisure satisfaction includes previous experience, individual expectation, achievement, or satisfaction perception in leisure activities. If the actual situation fails to meet the expectation, it will produce dissatisfaction, and when the actual situation meets the expectation, it will produce satisfaction. Therefore, leisure practitioners should understand that leisure activities are purpose-oriented behavior. How to achieve psychological, educational, social, relaxation, hygiene, and aesthetic satisfaction for different groups through leisure activities should be an important topic of health promotion in the future.
(2)Social capital and leisure behavior are interrelated, and this study constructs the causal process from the overall social structure factors in personal physical and mental health. Therefore, social capital is defined as an independent variable, leisure behavior as an intermediary variable, and health as a dependent variable. If future research supports leisure behavior as an independent variable and social capital as an intermediary variable, health as a dependent variable is an interesting research topic, which is worthy of further discussion by future scholars.

## Data Availability Statement

The original contributions presented in the study are included in the article/supplementary material, further inquiries can be directed to the corresponding author/s.

## Ethics Statement

This study was reviewed and approved by the Ethics Review Committee of North Sichuan Medical College. However, this study does not involve human and animal experiments, and written informed consent is not required.

## Author Contributions

XH was mainly responsible for the design of the paper, the preparation of the questionnaire, and participated in the writing of the paper. MH was mainly engaged in the distribution of the questionnaire and data processing and analysis. All authors contributed to the article and approved the submitted version.

## Conflict of Interest

The authors declare that the research was conducted in the absence of any commercial or financial relationships that could be construed as a potential conflict of interest.

## Publisher's Note

All claims expressed in this article are solely those of the authors and do not necessarily represent those of their affiliated organizations, or those of the publisher, the editors and the reviewers. Any product that may be evaluated in this article, or claim that may be made by its manufacturer, is not guaranteed or endorsed by the publisher.
